# Recent dengue virus infection: epidemiological survey on risk factors associated with infection in a medium-sized city in Mato Grosso

**DOI:** 10.1590/1516-3180.2020.0718.R1.18052021

**Published:** 2021-11-29

**Authors:** Dandára Thaís de Oliveira Ferreira, Marina Atanaka, Mariano Martinez Espinosa, Lavinia Schuler-Faccini, Aline da Silva Caldeira, Juliana Herrero da Silva, Viviane Karolina Vivi-Oliveira, Rayana de Castro da Paz, Vagner Ferreira do Nascimento, Ana Cláudia Pereira Terças-Trettel

**Affiliations:** I MSc. Nutritionist and Public Manager, Storage and Distribution Center for Medicines and Supplies of the Municipal Health Department, Várzea Grande (MT), Brazil.; II PhD. Nurse and Associate Professor IV, Postgraduate Program on Collective Health, Universidade Federal de Mato Grosso (UFMT), Cuiabá campus, Cuiabá (MT), Brazil.; III PhD. Statistician and Associate Professor IV, Postgraduate Program on Collective Health, Universidade Federal de Mato Grosso (UFMT), Cuiabá campus, Cuiabá (MT), Brazil.; IV MD, PhD. Full Professor, Department of Genetics, Universidade Federal do Rio Grande do Sul (UFRS), Porto Alegre (RS), Brazil.; V RN. Primary Healthcare Nurse, Municipal Health Department, Poconé (MT), Brazil.; VI MSc. Nurse and Technical Manager, Municipal Epidemiological Surveillance Department, Tangará da Serra (MT), Brazil.; VII MSc. Doctoral Student, Biologist and Professor, Department of Biomedicine, Universidade de Cuiabá, Cuiabá (MT), Brazil.; VIII Specialist. Pharmacist, Biochemist and Technical Consultant, General Coordination Office for Health Laboratories, Health Surveillance Department, Ministry of Health, Brasília (DF), Brazil.; IX PhD. Nurse and Adjunct Professor II, Universidade do Estado de Mato Grosso (UNEMAT), Tangará da Serra campus, Tangará da Serra (MT), Brazil.; X PhD. Nurse and Adjunct Professor, Universidade do Estado de Mato Grosso (UNEMAT), Tangará da Serra campus, Tangará da Serra (MT), Brazil; and Permanent Professor, Postgraduate Program on Collective Health, Universidade Federal de Mato Grosso (UFMT), Cuiabá campus, Cuiabá (MT), Brazil.

**Keywords:** Dengue, Risk factors, Health surveys, Serology, Health education, Arboviral disease, Socioeconomically disadvantaged, Epidemiological behavior, Sanitary conditions

## Abstract

**BACKGROUND:**

Dengue is considered to be the most important arbovirus worldwide, with important complications that increase its lethality. In Brazil, an endemic country, the disease reaches significant incidence levels, with occurrences of serious cases and high costs of hospitalizations for its treatment.

**OBJECTIVE:**

To analyze risk factors among individuals with recent histories of dengue infection in a medium-sized city in Mato Grosso.

**DESIGN AND SETTING:**

Descriptive cross-sectional study, of epidemiological-survey type, conducted among the urban population of a city located in mid-northern Mato Grosso.

**METHODS:**

A seroepidemiological survey using questionnaires and collection of biological material was conducted among 596 adults aged ≥ 18 years who had been selected through a cluster sampling process. Positive dengue cases were those with positive results from anti-dengue immunoassays (ELISA). Statistical analyses with descriptive and inferential techniques were used, with 95% confidence intervals and a 5% significance level.

**RESULTS:**

The seroepidemiological profile of the study participants was predominantly female, with ages between 18 and 39 years, self-declared non-white race/color, not more than eight years of education and not living with a companion. Among the sanitary factors analyzed, the following were risk factors for dengue virus infection: no running water at home; no water supply from the public piped network; no waste from drains or toilets sent to the sewage network; endemic disease combat agents visiting the home; and presence of mosquito breeding sites at home.

**CONCLUSION:**

Low schooling levels and previous dengue virus infection were associated with current dengue virus infection.

## INTRODUCTION

Dengue has been shown to be one of the most important urban arboviruses. It is considered to constitute a reemerging infectious epidemic of great magnitude, responsible for morbidity and mortality among millions of people in more than 125 countries. In 2019, 2.7 million cases were recorded in the Americas, of which 22,127 were of great severity, and 1,206 deaths were reported. In Brazil, in the same year, there were 1,544,987 suspected cases of dengue and an incidence rate of 735.2 cases per 100,000 inhabitants. That year has been characterized as the one with the highest incidence in the history of this disease.^1,2,3,4,5^

Viral variations contribute to increased numbers of cases and especially to occurrences of severe forms of the disease. Four antigenically distinct serotypes of dengue are now circulating simultaneously, which enables infection by all variants. Thus, the clinical manifestations of this disease are diverse: it may be asymptomatic or may give rise to alterations in cell permeability, multiple organ failure and even death. Some complications may be present, such as neurological alterations, secondary bacterial infections, cardiac and respiratory dysfunctions and rhabdomyolysis.^6,7,8,9^

Knowing the incidence of the disease is essential for direct prevention and monitoring actions. As shown by the main population-based studies conducted in all regions of Brazil since 1999, the incidence of dengue virus infection has ranged from 4.0 to 90.1%.^10,11,12,13,14,15,16,17,18^ This variation has mainly been due to the local circulating viral serotype and year of study. The central-western region of Brazil is ranked second highest for dengue cases in this country, and the average incidence rate in the state of Mato Grosso is 30%.^19,20,21^

Mato Grosso has high temperatures and low humidity. It has a diversity of environments, comprising areas of the Cerrado, Pantanal and Amazon biomes, which are undergoing constant environmental transformation. These factors may favor occurrences of human infections caused by numerous pathogens.^21,22^

## OBJECTIVE

In the light of this scenario, the aim of the present study was, through an epidemiological survey, to analyze risk factors among individuals with recent histories of dengue infection in a medium-sized city in Mato Grosso.

## METHODS

This was a descriptive and cross-sectional study, of epidemiological-survey type, conducted among the urban population of a city located in mid-northern Mato Grosso. Data collection was performed between January and March 2018, by a previously trained team, and a pilot study was first conducted in a census tract that did not form part of the final sampling.

**Figure 1.**
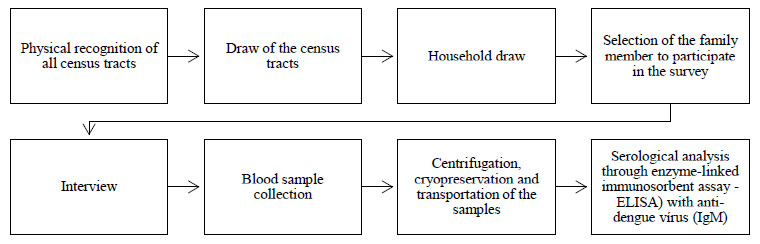
Sample selection from the dengue epidemiological survey.

A cluster sampling process was carried out in two stages (census tracts and households). Initially, these tracts (delimited by the Brazilian Institute for Geography and Statistics) were assessed to identify inhabited homes (households), i.e. to determine whether these tracts contained households, shops, wasteland or other types of land use, as shown in Figure 1. From this, an updated listing of census tracts was obtained. Proportional numbers of households were then systematically intercalated for each census tract sampled.

To estimate the serological prevalence of dengue in the population, with a margin of error of five percentage points, a sample of 660 individuals was required. Thus, a standard error of 0.025 was adopted for a sample of 400 individuals, and 10% loss was added to this number, plus inflation of 1.5, to reach this total of 660 individuals.

The study included individuals aged 18 years or older who were already living in the urban area of this city before April 1, 2016, and who remained as residents until the time of data collection in 2018. Women of childbearing age and pregnant women were also included. Institutionalized individuals were excluded from the study. Among the 660 individuals drawn, 64 left the study due to refusal or absence. Thus, the final sample for the study comprised 596 individuals. Selection of these individuals was carried out by means of simple random sampling among the residents of the selected households. Data collection only began after the potential participants had signed an informed consent statement.

Data collection was performed in the households, and took approximately 40 minutes per household. The interview consisted of application of a structured questionnaire, composed of questions that addressed sociodemographic, health and sanitary factors.

The dependent variable of this study was recent seropositivity for dengue (immunoglobulin M, IgM). The independent variables analyzed were sex, age group, schooling, race/color, marital status, type of household, running water, main form of water supply, bathroom drain, garbage destination, use of mosquito nets, visits by endemic disease combat agents, presence of mosquito breeding sites and hospitalization due to dengue.

For serological analysis, peripheral venipuncture was performed in an antecubital region, to collect 6 ml of blood in a vacuum collection tube containing separator gel. These samples were then sent to the laboratory of the city’s Epidemiological Surveillance Department. There, they were centrifuged and cryopreserved in order to transport them to the Virology Laboratory of the Medical School of the Federal University of Mato Grosso in Cuiabá, Mato Grosso, to be kept in an ultra-freezer (-80 °C).

Serological analyses were performed at the Central Public Health Laboratory of Mato Grosso, in Cuiabá. These were done in accordance with biosafety standards, through an immunoenzymatic assay (ELISA) using a IgM dengue virus kit (batch: ESR114M; Virion Serion, Wolfsburg, Germany).

The interview responses and laboratory results were double-entered, using a form constructed in the EpiInfo software, version 7 for Windows (CDC, Atlanta, Georgia, United States). Subsequently, the data were checked for any inconsistencies, using the Excel 365 software for Windows (Microsoft Corporation, Redmond, Washington, United States), thus originating the final database for analysis. For data processing, the IBM Statistical Package for the Social Sciences (SPSS), version 20.0 (IBM Corporation, Armonk, New York, United States) was used.

Descriptive and inferential statistical techniques were used for the analyses. In the descriptive analyses, proportions and tables were used, while in the inferential analyses, chi-square tests, Fisher’s exact test and crude prevalence ratios were obtained, with their respective 95% confidence intervals (CI). For all inferences, the significance level was taken to be 5%. For multiple analysis, the Poisson regression model was used, in which the independent variables were introduced into the method using a backward procedure. All variables that presented P < 0.20 in bivariate analyses were used in building the multiple model. For the final model, the variables that continued to present P-values below 0.05 (P < 0.05) were retained.

The confidentiality of the information provided by each participant was guaranteed. It was emphasized to the participants that all results would be handed over to them. Individuals whose results confirmed the presence of the disease were met by the municipal healthcare team, duly notified and brought into the flow of investigation and clinical management of dengue.

This study formed part of a matrix project that was approved by the Research Ethics Committee of the Clinical Hospital of Porto Alegre under opinion report no. 2.068.222, with Certificate of Presentation for Ethical Analysis no. 56176616.2.1001.5327, approved on May 17, 2017.

## RESULTS

The sociodemographic profile of the 596 study participants was predominantly female (67.8%), of whom 45.5% were aged 18 to 39 years, 58.4% had had not more than eight years of schooling, 67.2% had non-white ethnicity/color and 51.1% said that they did not live with an affective companion.

Among those with recent infection detected through a serological test (IgM), the seroepidemiological profile of the study participants was composed of females (26.7%), aged between 18 and 39 years (28.2%), with not more than eight years of schooling (29.1%), non-white race/color (26.3%), who did not live with an affective companion (28.3%), as shown in Table 1.

**Table 1.** Prevalences and 95% confidence intervals of dengue cases, according to sociodemographic variables in a city in Mato Grosso, Brazil, 2018

Variable	n (%)	Positive	Negative	cPR	95% CI	P-value
		n (%)	n (%)			
**Sex**						
Male	192 (32.2%)	47 (24.5%)	145 (75.5%)	0.92	(0.68-1.23)	0.558
Female	404 (67.8%)	108 (26.7%)	296 (73.3%)	1.00	–	–
**Age group (years)**						
18 to 39	271 (45.5%)	53 (28.2%)	135 (71.8%)	0.92	(0.50-1.67)	0.780
40 to 59	201 (33.7%)	66 (25.0%)	198 (75.0%)	1.13	(0.83-1.54)	0.451
≥ 60	124 (20.8%)	14 (12.0%)	103 (88.0%)	1.00	–	–
**Schooling (years)**						
Illiterate/≤ 8	247 (41.6%)	72 (29.1%)	175 (70.9%)	1.23	(0.94-1.62)	0.130
> 8	347 (58.4%)	82 (23.6%)	265 (76.4%)	1.00	–	–
**Race/color**						
Non-white	399 (67.2%)	105 (26.3%)	294 (73.7%)	1.07	(0.79-1.44)	0.656
White	195 (32.8%)	48 (24.6%)	147 (75.4%)	1.00	–	–
**Living with a companion**						
No	300 (51.1%)	85 (28.3%)	215 (71.7%)	1.23	(0.92-1.60)	0.168
Yes	287 (48.9%)	67 (23.3%)	220 (76.7%)	1.00	–	–

cPR = crude prevalence ratio; 95% CI = 95% confidence interval. n: sample size according to variable; P: chi-square test.

The seroprevalence profile of dengue virus infection (DENV) according to sociosanitary characteristics and history of disease (Table 2), as constructed from bivariate analyses, had the following characteristics: individuals living in apartments (crude prevalence ratio, cPR: 0.77; 95% CI: 0.37-1.60; P < 0.552 from Fisher’s exact test); without running water (cPR: 1.30; 95% CI: 0.42-4.06; P < 0.650 from Fisher’s exact test); whose water supply did not come through the general [piped] distribution network (cPR: 1.21; 95% CI: 0.78-1.89; P < 0.413); whose wastewater disposal from the bathroom drain was to a destination other than the sewage network (cPR: 1.21; 95% CI: 0.85-1.73; P < 0.276); and whose garbage was removed by the public garbage collection service (cPR: 0.58; 95% CI: 0.29-1.16; P < 0.095).

**Table 2.** Prevalences and 95% confidence intervals of positive and negative dengue cases (immunoglobulin M, IgM), according to health variables and disease history in a city in Mato Grosso, Brazil, 2018

Variable	Positive	Negative	cPR	95% CI	P-value
	n (%)	n (%)			
**Type of household**					
House	148 (25.8%)	426 (74.2%)	0.77	(0.37-1.60)	0.552*
Apartment	5 (33.3%)	10 (66.7%)	1.00	–	–
**Running water**					
No	2 (33.3%)	4 (66.7%)	1.30	(0.42-4.06)	0.650*
Yes	150 (25.6%)	435 (74.4%)	1.00	–	–
**Main form of water supply**					
Other	15 (30.6%)	34 (69.4%)	1.21	(0.78-1.89)	0.413
General network	137 (25.3%)	405 (74.7%)	1.00	–	–
**Bathroom drain**					
Other	125 (27.1%)	337 (72.9%)	1.21	(0.85-1.73)	0.276
Sewage network	29 (22.3%)	101 (77.7%)	1.00	–	–
**Destination of garbage**					
Other	7 (15.6%)	38 (84.4%)	0.58	(0.29-1.16)	0.095
Public garbage collection service	148 (26.9%)	402 (73.1%)	1.00	–	–
**Use of mosquito net**					
No	143 (26.2%)	402 (73.8%)	1.05	(0.63-1.75)	0.851
Yes	12 (25.0%)	36 (75.0%)	1.00	–	–
**Visit by endemic disease combat agents**					
Every 2 to 4 months	50 (31.1%)	111 (68.9%)	1.22	(0.86-1.72)	0.257
Once	15 (20.8%)	57 (79.2%)	0.82	(0.49-1.38)	0.443
Don’t remember/don’t know	45(23.8%)	144 (76.2%)	0.94	(0.65-1.34)	0.720
Monthly	43 (25.4%)	126 (74.6%)	1.00	–	–
**Breeding site for mosquitoes was found**					
Yes	14 (28.0%)	36 (72.0%)	1.05	(0.65-1.69)	0.849
No	93 (26.7%)	255 (73.3%)	1.00	–	–
Unknown /not applicable	48(24.5%)	148 (75.5%)	0.92	(0.68-1.24)	0.568
**Hospitalization due to dengue**					
Yes	7 (12.5%)	49 (87.5%)	0.49	(0.20-1.19)	0.005
No	61 (31.6%)	132 (68.4%)	1.00	–	–

cPR = crude prevalence ratio; 95% CI = 95% confidence interval. n: sample size according to variable; P: chi-square test; *Fisher’s exact test.

Among the participants who were positive for DENV, 26.2% reported not using a mosquito net, 31.1% had had visits from endemic disease combat agents every two to four months and 28.0% had already found mosquito-breeding sites in their homes. Although all of these last variables mentioned were not statistically significant for DENV infection, they were shown to be risk factors for infection. Regarding the participants’ self-reported histories of the disease, the variable of hospitalization due to dengue presented a statistically significant association with DENV infection. In this study, the overall prevalence of DENV infection (defined as IgM) in the population was 26.0%.

In the final model obtained through robust Poisson regression (rPR), the variable of hospitalization due to dengue (cPR: 1.30; 95% CI: 1.01-1.69; P < 0.014, i.e. significant at the 5% level) maintained statistical significance with DENV infection, whereas the variable of schooling (years of education) (cPR: 1.49; 95% CI: 1.04-1.49; P < 0.047, i.e. significant at the 5% level) became statistically significant only in this final model (Table 3).

**Table 3.** Variables in the final model and prevalence ratios adjusted using robust Poisson regression (rPR) that presented associations with positive and negative dengue cases (immunoglobulin M, IgM), with their respective 95% confidence intervals (CI) and P-values, in a city in Mato Grosso, Brazil, 2018

Variable	Category	rPR	95% CI	P-value
**Hospitalization due to dengue**	Yes	1.30	1.01-1.69	0.014*
	No	1.00	–	–
**Schooling (years)**	Illiterate/≤ 8	1.49	1.04-1.49	0.047*
	> 8	1.00	–	–

rPR = prevalence ratio adjusted using robust Poisson regression model with variable selection by means of backward method. CI: confidence interval; *Significant at the level of 5%. Note: The garbage destination variable remained in the model as the adjustment variable, although the P-value was greater than 0.05.

## DISCUSSION

In the city investigated, from 2008 to January 2020, according to the epidemiological bulletin of diseases transmitted by the vector *Aedes aegypti* that was issued by the city’s Epidemiological Surveillance Department, 7,581 suspected cases of dengue were reported among residents. Of these, 5,933 cases were confirmed, with a higher average endemic index between January and May of each year. In the epidemiological survey conducted in 2018, which was conducted during this period of higher endemic index, in this city, 155 cases of dengue were confirmed by means of serological tests (IgM) among the 596 participants. Thus, the overall prevalence of DENV infection among the participants was 26.0%.

Regarding the seroprevalence found, similar studies have been conducted in several countries to test their populations for recent infection (IgM) by DENV. This was done among individuals over the age of 15 in a dengue hyperendemic area in Barranquilla, Colombia,^23^ among children and adolescents under 18 years and adults of ages between 18 and 95 years old in seven municipalities of five provinces in Colombia,^24^ among adults in Jamaica^25^ and among individuals with suspected dengue fever in southern Odisha^26^ in India and Khyber Pakhtunkhawa^27^ in Pakistan, and the percentage prevalences found were 14.9%, 11.8%, 3.6%, 21.05% and 31.86%, respectively. The prevalence of DENV found in the population in southern Odisha, India (21.05%) was the closest to what was found in the present study (26.0%).

Regarding the percentages of seroprevalence, it is worth mentioning the possibility of occurrences of cross-reactivity in serological tests between DENV, Zika virus (ZIKV) and chikungunya virus (CHIKV). These arboviruses all circulate simultaneously in the city of the present study in Mato Grosso. DENV and ZIKV have already caused epidemics in previous years in this city (DENV in 2009, 2012 and 2013; ZIKV in 2016). CHIKV has circulated in this city since 2016 and had its peak of cases in 2018, i.e. before the time of data collection for the present survey.^28^ In this context of cross-reactivity, there is protein homology between DENV and ZIKV, which are both flaviviruses and share all the essential structural characteristics, such as capsids, envelopes, membrane protein precursors and quaternary structures. This consequently enables considerable immunological cross-reactivity.^29,30,31,32^ Simultaneous detection of antibodies to DENV and CHIKV is also observed, and these can respond either through cross-reactivity or through coinfection.^33^

Although the municipality studied here and Odisha, in India, had similar prevalences, which were both higher among women, the prevalences according to age groups were different: in Tangará, cases were concentrated between the ages of 18 and 39 years (28.2%), while in Odisha, the largest proportion was between 11 and 20 years of age (42.5%).

There were no statistically significant differences in any of the sociodemographic variables in the present study, except for schooling. Although schooling levels did not present any significant differences in the bivariate analysis (cPR: 1.23; 95% CI: 0.92-1.62; P < 0.130), it was found in the final model that being illiterate or having not more than eight years of education was correlated with a risk of DENV infection (rPR: 1.49; 95% CI: 1.04-1.49; P < 0.047, i.e. significant at the 5% level) (Table 3).

The association between low schooling level and occurrences of DENV infection that was observed in the city of the present study has also been one of the sociodemographic factors correlated with positive DENV cases elsewhere. However, this was not as an isolated factor, but as part of a larger set of sociodemographic, economic and poor health factors that influence occurrences of dengue cases. Schooling has been pointed out as an important aspect of overall awareness levels regarding dengue. This awareness includes knowing about the vectors that transmit the disease, identifying and implementing disease control and prevention methods within the population and recognizing the signs and symptoms of the disease process.^34,35,36,37^

Individuals with low levels of education were also among those with lower levels of knowledge about the disease. Their situation was the inverse of people who had attended college/university and who, therefore, formed part of the group with the highest percentage of seronegativity for DENV.^38^ A study conducted in Sri Lanka on the level of awareness of dengue among schoolchildren (13 to 15 years of age) recommended that educational programs aimed at raising awareness and knowledge of dengue prevention and control practices should be included within teaching of young populations, in order to contribute to transformation of knowledge into good practices against DENV in endemic areas and to help in controlling future epidemics.^37^

Investigations on the association of schooling levels with DENV infection have also portrayed how health and/or environmental factors can influence exposure to infection. Regarding these factors and the risk of DENV infection, scenarios that favor infection involve the type of housing of the population and the regularity and means of obtaining and using water. Thus, inadequate storage of water, inadequate sewage disposal and insufficient urban garbage collection, in addition to little or no practical action aimed at vector prevention, favor DENV transmission.^34,36,38,39,40,41,42^ Nonetheless, in the present study, there were no statistically significant associations for any of the health variables analyzed, as was also previously observed in studies conducted in the city of Belo Horizonte (Minas Gerais) in 2008 and in the city of Caraguatatuba (São Paulo) in 2018.^40,43^

Directly associations with risks of DENV infection were observed with regard to nonuse of protective measures such as mosquito nets (26.2%), presence of mosquito breeding sites (28.0%) and visits by endemic disease combat agents (31.1%) to homes at intervals of two to four months. These would constitute preventive measures against the transmitting vectors.

One important part of the actions to combat the vectors that transmit DENV is health education actions. However, the dengue control model in Brazil remains political and case-anchored, which includes occasional investments in campaigns against mosquitoes and guidance on actions through the media, with little effective impact on the fight against the disease in general.^44,45^ Traditionalism in the control model is characterized by lack of innovation in the strategies to cope with endemic disease, combined with little improvement in infrastructure and little investment in new means of empowering healthcare professionals or embracing the community.^3,44,46^ A study conducted in Icaraí-Caucaia (Ceará) on health education for dengue prevention and control concluded that health education was delivered ineffectively, without dialogue between healthcare professionals and the population, and that generalist methods of knowledge transmission by healthcare professionals were fruitless in combating a disease such as dengue.^45^

Although dengue is a debilitating and self-limiting disease, most patients follow a benign clinical course and recover. However, some cases may evolve to severe forms and death. Dengue can present three clinical phases, i.e. febrile, critical and recovery, which highlights the need for attention to and monitoring of the appearance of warning signs. These may indicate evolution to the severe forms of the disease, which would usually appearing on the third to seventh day after the onset of the disease. Dengue with complications is characterized by severe bleeding, severe organ dysfunction or significant plasma extravasation, with additional severity in children and the elderly, because they present a faster disease course and are vulnerable to complications inherent to their ages.^2^ Some studies have also indicated the possibility of mutual increment of infections between DENV and ZIKV from extrinsic improvement dependent on antibodies. This is a phenomenon that has been implicated in severe forms of dengue, such as dengue hemorrhagic fever and dengue shock syndrome, and in cases of increased severity of the disease through secondary infection by DENV.^29,30^

Hospitalization can be indicated for both forms, i.e. dengue hemorrhagic fever and dengue shock syndrome. The objective is to maintain hydration and hydroelectrolytic balance.^47,48^ Hospitalization due to dengue was one of the variables that were statistically associated (cPR: 1.30; 95% CI: 1.01-1.69; P < 0.014, i.e. significant at the 5% level) (Table 3) with DENV infection and with serological tests (IgM) that identified recent infection in individuals. This may be a secondary response to previous infection by other flaviviruses or other DENV serotypes.^2,29,30,31,32,33,48^ This highlights that the results from the present study may reflect cross-reactivity between DENV and ZIKV, since it was conducted two years after an epidemic of ZIKV.

Over the last decade, 546,939 hospitalizations due to dengue treatment were recorded in Brazil, with a total cost of 184 million reais. The central-western region occupied third place among the five geographical regions of Brazil, both in numbers of hospitalizations (91,540) and in hospitalization expenditure (30,366,167.67).^49^ Dengue is known to be a serious public health problem that generates high costs for this country’s population and economy. It negatively affects individuals, families and social productivity.^50,51,52^ Therefore, putting health surveillance actions into practice, while considering the individual characteristics of each population, listening to the community and providing training for healthcare professionals, is an important form of contributing to coping with diseases of great magnitude such as dengue.

It is of paramount importance to know the factors that are associated with DENV infection, given its already-known association with social vulnerability, and also its impact on society as a whole. The present study is important insofar as it provides knowledge of yet another dengue distribution profile, in a medium-sized city in a state covering an immense area.

The following limitations of the present study need to be taken into consideration. Children (under the age of 18 years) were not included in this survey, given that children, along with the elderly, make up the risk group for dengue cases. The serotypes circulating among the participants were not identified, although this is necessary in order to ascertain how the disease is circulating in the community. No other analyses such as the reverse transcriptase polymerase chain reaction (RT-PCR) were performed to confirm recent cases of dengue, although that could also have helped in identifying cross-reactivity between DENV and the Zika and chikungunya arboviruses that circulate simultaneously with dengue in this city.

## CONCLUSIONS

In this cross-sectional study conducted in a city located in mid-northern Mato Grosso, the findings showed that there were no significant associations between DENV infection and the sanitary variables that characterized the population. However, some variables were risk factors for infection. The variables of hospitalization due to dengue and schooling level were statistically significant with regard to occurrences of DENV infection, in the final model. This may indicate the existence of a relationship between low schooling levels and lower awareness of how to protect oneself against the infection and how to prevent it.

The data from the present study allowed us to identify the profile of recent dengue infection in this city, thereby generating information about the population that provides an analysis on the population’s health situation. The results obtained raise the possibility of conducting further studies to determine the serotypes of DENV, as well as to investigate occurrences of cross-reactivity with other arboviruses circulating in this city.
